# Supplementary material for: Activin A Promotes Osteoblastic Differentiation of Human Preosteoblasts through the ALK1-Smad1/5/9 Pathway
Source: Int J Mol Sci. 2021 Dec 16;22(24):13491. doi: 10.3390/ijms222413491 (PMC8704413; doi:10.3390/ijms222413491)
Supplement: Supplementary file 1 [file ijms-22-13491-s001.zip › ijms-1451548-supplementary.pdf]

Supplemental Figures S1-S9

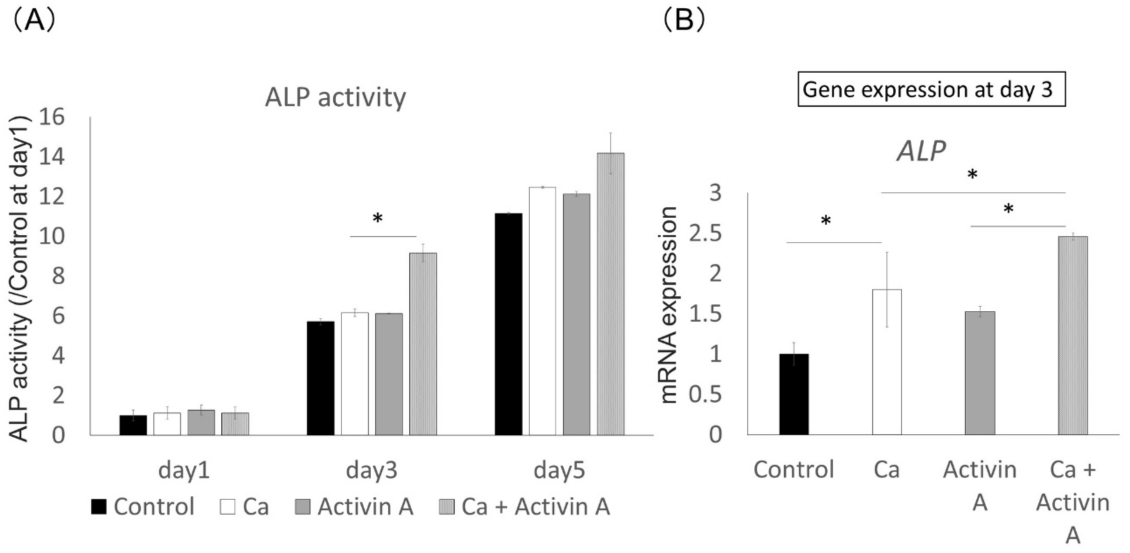

Supplemental Figure S1 ALP activity in Saos2 cells treated with Activin A

(A) Saos2 cells treated with 0.5 mM $CaCl_2$ (Ca) and 100 ng/ml Activin A were cultured for 1, 3, and 5 days. ALP activity assay was performed using ALP assay kit at an absorbance of 405 nm. Untreated cells were used as the control and the value was shown as the fold-increase of the control at day1. (B) Gene expression of *ALP* in Saos2 cells treated with 0.5 mM $CaCl_2$ and 100 ng/ml Activin A for 3 days was analyzed using quantitative RT-PCR. Untreated cells were used as the control. Normalization of gene expression was performed against *β-actin* expression, and the gene expression levels were shown as the fold-increase of the control. Values were the means ± SD from three independent experiments. *P < 0.05.

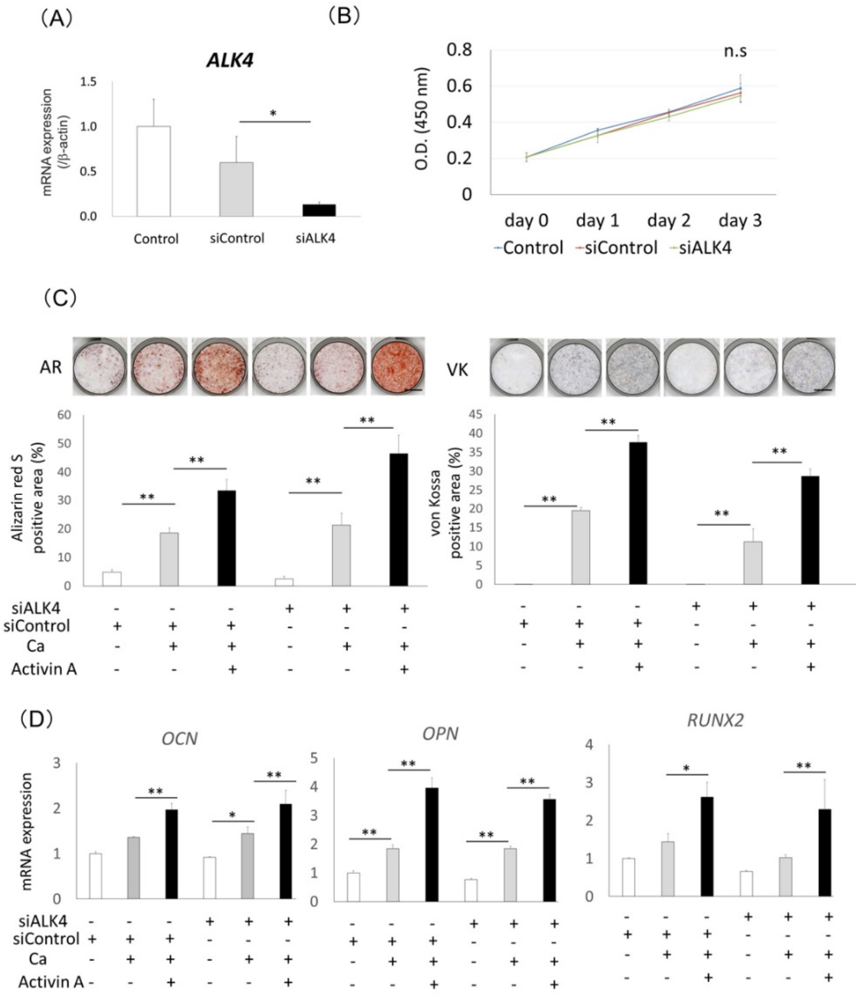

Supplemental Figure S2 Treatment with siALK4 exhibited no effect on Activin A-induced osteoblastic differentiation of Saos2 cells

(A) Gene expression of *ALK4* in Saos2 cells treated with siALK4. Untreated cells were used as the control. Gene expression of *ALK4* was examined using quantitative RT-PCR. Gene expression levels were normalized against *β-actin* expression, and the results are shown as the fold-increase of the control. Values are the means ± SD from three independent experiments. *$P < 0.05$. (B) Saos2 cells treated with siALK4 were cultured in 10%FBS/αMEM for 0, 1, 2, and 3 days. A proliferation assay was performed using a WST-1 proliferation assay kit at an absorbance of 450 nm. (C) Saos2 cells were cultured with 0.5 mM $CaCl_2$ (Ca) with or without 100 ng/ml Activin A. Mineralization was visualized by Alizarin Red S staining (AR) and von Kossa staining (VK), and the staining area was quantitated. **$P < 0.01$. (D) Expression of bone-related genes (*OCN*, *OPN,* and *RUNX2*) in Saos2 cells treated with 100 ng/ml Activin A was examined by quantitative RT-PCR. Untreated cells were used as a control. The expression levels of these genes were normalized against *β-actin* expression, and the results are shown as the fold-increase of the control. *$P < 0.05$, **$P < 0.01$.

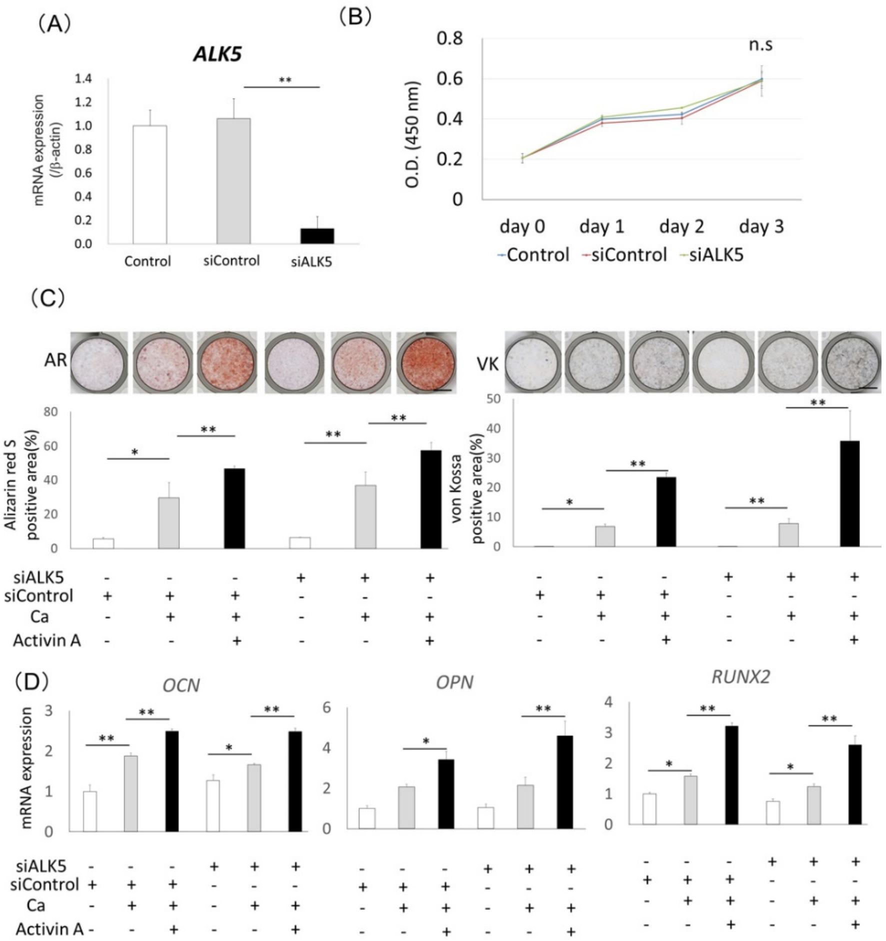

Supplemental Figure S3 Treatment with siALK5 exhibited no effect on Activin A-induced osteoblastic differentiation of Saos2 cells

(A) Gene expression of *ALK5* in Saos2 cells treated with siALK5. Untreated cells were used as the control. Gene expression of *ALK5* was examined using quantitative RT-PCR. Gene expression levels were normalized against *β-actin* expression, and the results are shown as the fold-increase of the control. Values are the means ± SD from three independent experiments. **P < 0.01. (B) Saos2 cells treated with siALK5 were cultured in 10%FBS/αMEM for 0, 1, 2, and 3 days. A proliferation assay was performed using a WST-1 proliferation assay kit at an absorbance of 450 nm. (C) Saos2 cells were cultured with 0.5 mM $CaCl_2$ (Ca) with or without 100 ng/ml Activin A. Mineralization was visualized by Alizarin Red S staining (AR) and von Kossa staining (VK), and the staining area was quantitated. *P < 0.05, **P < 0.01. (D) Expression of bone-related genes (*OCN*, *OPN*, and *RUNX2*) in Saos2 cells treated with 100 ng/ml Activin A was examined by quantitative RT-PCR. Untreated cells were used as a control. The expression levels of these genes were normalized against *β-actin* expression, and the results are shown as the fold-increase of the control. *P < 0.05, **P < 0.01.

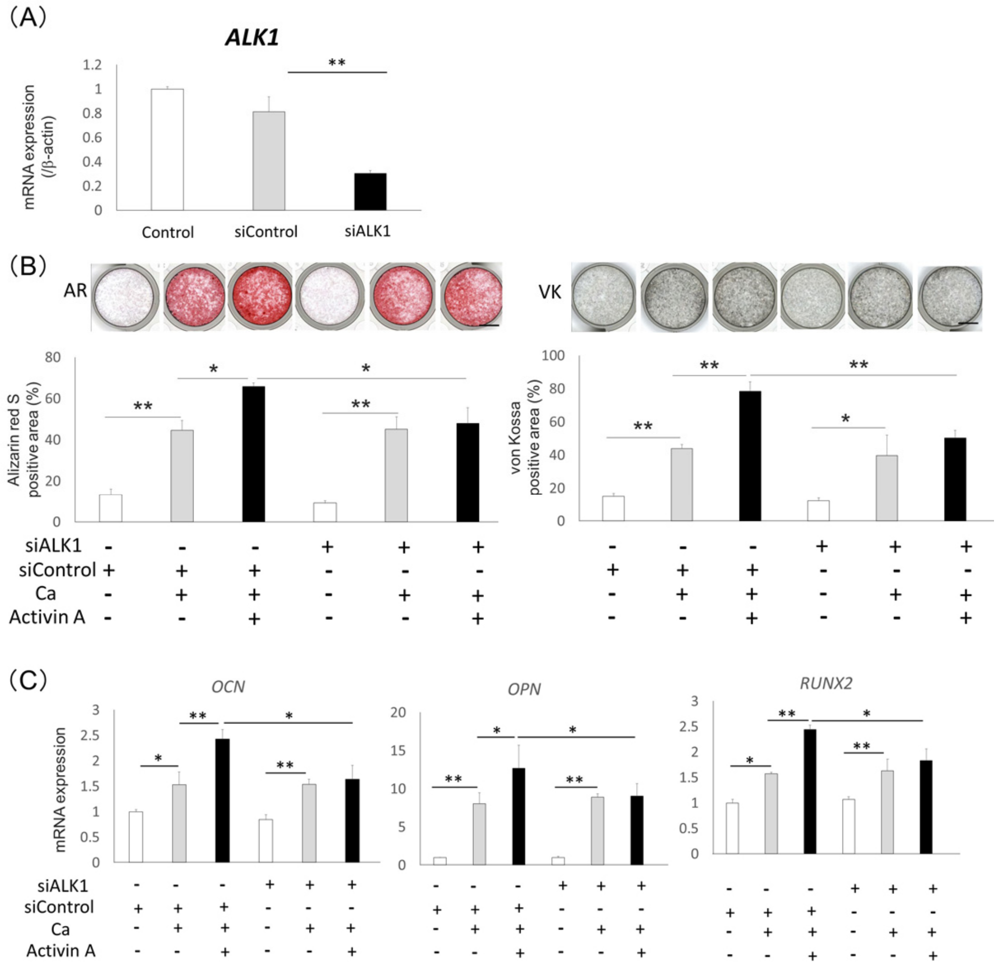

Supplemental Figure S4 Treatment with siALK1 inhibited Activin A-induced osteoblastic differentiation of NOS1 cells

(A) Gene expression of *ALK1* in NOS1 cells with siALK1. Untreated cells were used as the control. Gene expression of *ALK1* was analyzed using quantitative RT-PCR. Normalization of gene expression was performed against *β-actin* expression, and the gene expression levels were shown as the fold-increase of the control. Values are the means ± SD from three independent experiments. **$P < 0.01$. (B) NOS1 cells were cultured with 0.5 mM $CaCl_2$ (Ca) with or without 100 ng/ml Activin A. Mineralization of NOS1 cells was evaluated by Alizarin Red S staining (AR) and von Kossa staining (VK), and quantification of the staining area was analyzed. *$P < 0.05$, **$P < 0.01$. (C) Expression of bone-related genes (*OCN*, *OPN*, and *RUNX2*) in NOS1 cells treated with 100 ng/ml Activin A was analyzed by quantitative RT-PCR. Untreated cells were used as a control. Normalization of gene expression was performed against *β-actin* expression, and the gene expression levels were shown as the fold-increase of the control. *$P < 0.05$, **$P < 0.01$.

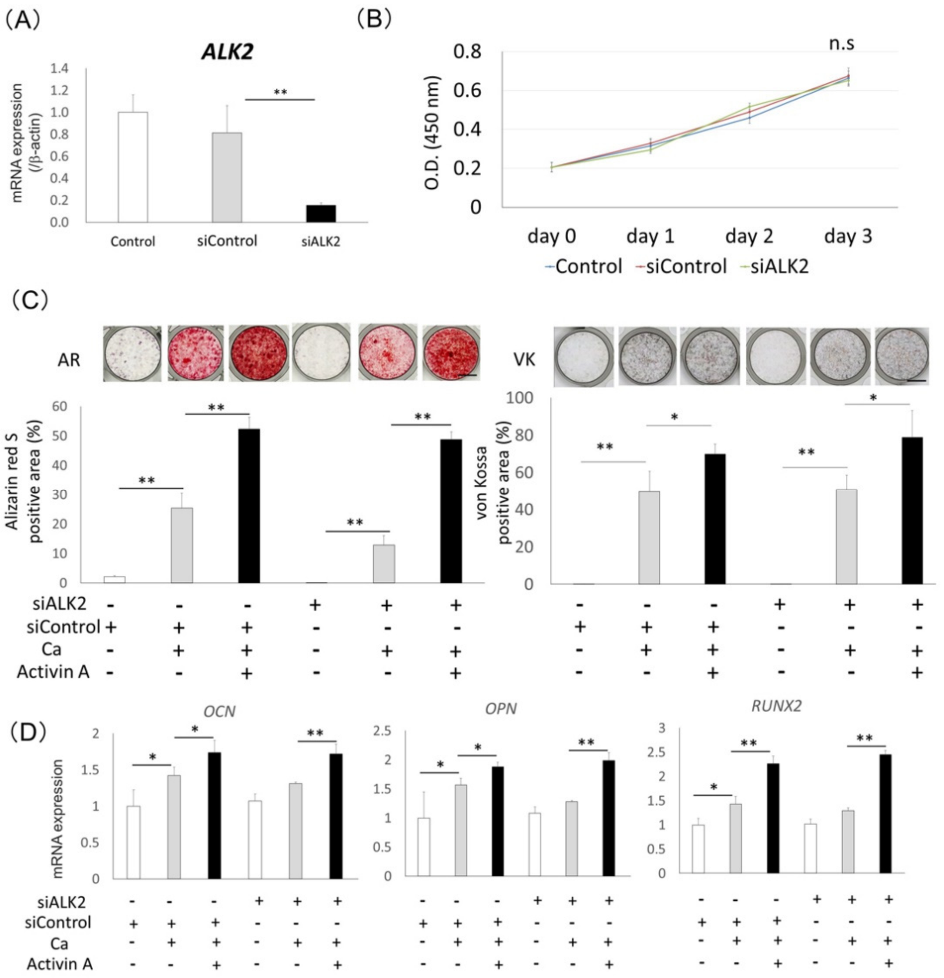

Supplemental Figure S5 Treatment with siALK2 had no effect on Activin A-induced osteoblastic differentiation of Saos2 cells

(A) Gene expression of *ALK2* in Saos2 cells treated with siALK2. Untreated cells were used as the control. Gene expression of *ALK2* was examined using quantitative RT-PCR. Gene expression levels were normalized against *β-actin* expression, and the results are shown as the fold-increase of the control. Values are the means ± SD from three independent experiments. **P < 0.01. (B) Saos2 cells treated with siALK2 were cultured in 10%FBS/αMEM for 0, 1, 2, and 3 days. A proliferation assay was performed using a WST-1 proliferation assay kit at an absorbance of 450 nm. (C) Saos2 cells were cultured with 0.5 mM $CaCl_2$ (Ca) with or without 100 ng/ml Activin A. Mineralization was visualized by Alizarin Red S staining (AR) and von Kossa staining (VK), and the staining area was quantitated. *P < 0.05, **P < 0.01. (D) Expression of bone-related genes (*OCN*, *OPN*, and *RUNX2*) in Saos2 cells treated with 100 ng/ml Activin A was examined by quantitative RT-PCR. The expression levels of these genes were normalized against *β-actin* expression, and the results are shown as the fold-increase of the control. *P < 0.05, **P < 0.01.

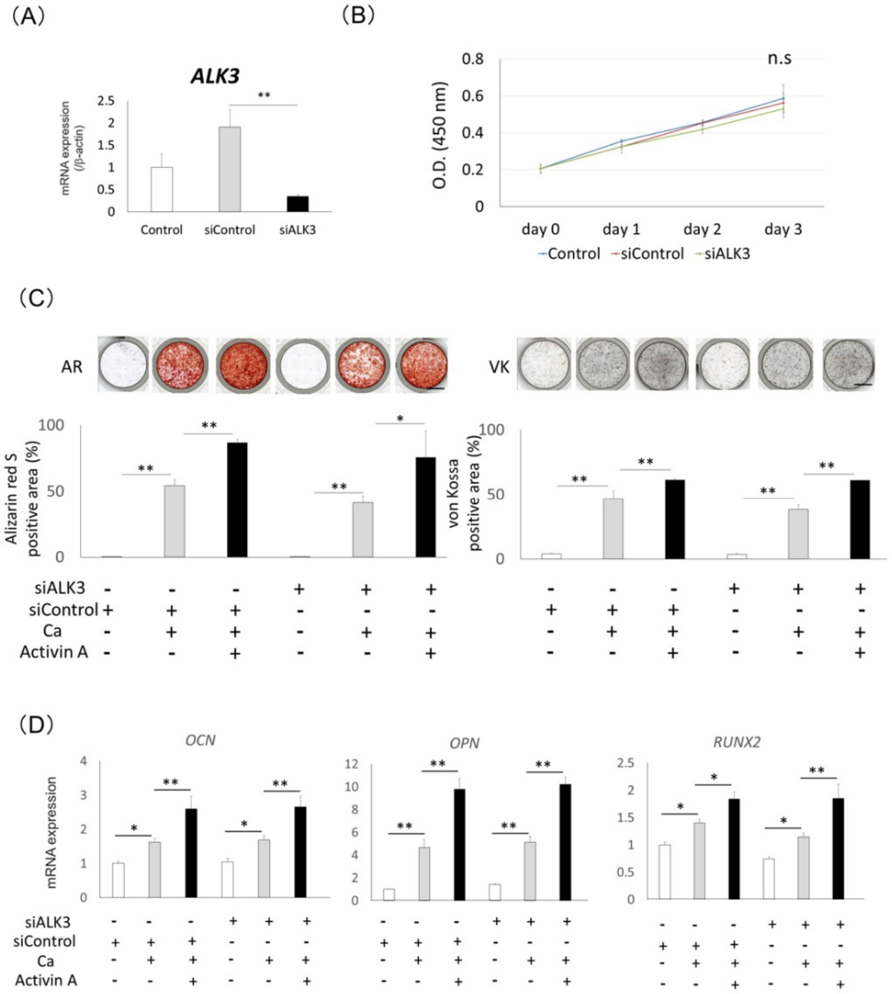

Supplemental Figure S6 Treatment with siALK3 had no effect on Activin A-induced osteoblastic differentiation of Saos2 cells

(A) Gene expression of *ALK3* in Saos2 cells treated with siALK3. Untreated cells were used as the control. Gene expression of *ALK3* was examined using quantitative RT-PCR. Gene expression levels were normalized against *β-actin* expression, and the results are shown as the fold-increase of the control. Values are the means ± SD from three independent experiments. **P < 0.01. (B) Saos2 cells treated with siALK3 were cultured in 10%FBS/αMEM for 0, 1, 2, and 3 days. A proliferation assay was performed using a WST-1 proliferation assay kit at an absorbance of 450 nm. (C) Saos2 cells were cultured with 0.5 mM $CaCl_2$ (Ca) with or without 100 ng/ml Activin A. Mineralization was visualized by Alizarin Red S staining (AR) and von Kossa staining (VK), and the staining area was quantitated. *P < 0.05, **P < 0.01. (D) Expression of bone-related genes (*OCN*, *OPN*, and *RUNX2*) in Saos2 cells treated with 100 ng/ml Activin A was examined by quantitative RT-PCR. Untreated cells were used as a control. The expression levels of these genes were normalized against *β-actin* expression, and the results are shown as the fold-increase of the control. *P < 0.05, **P < 0.01.

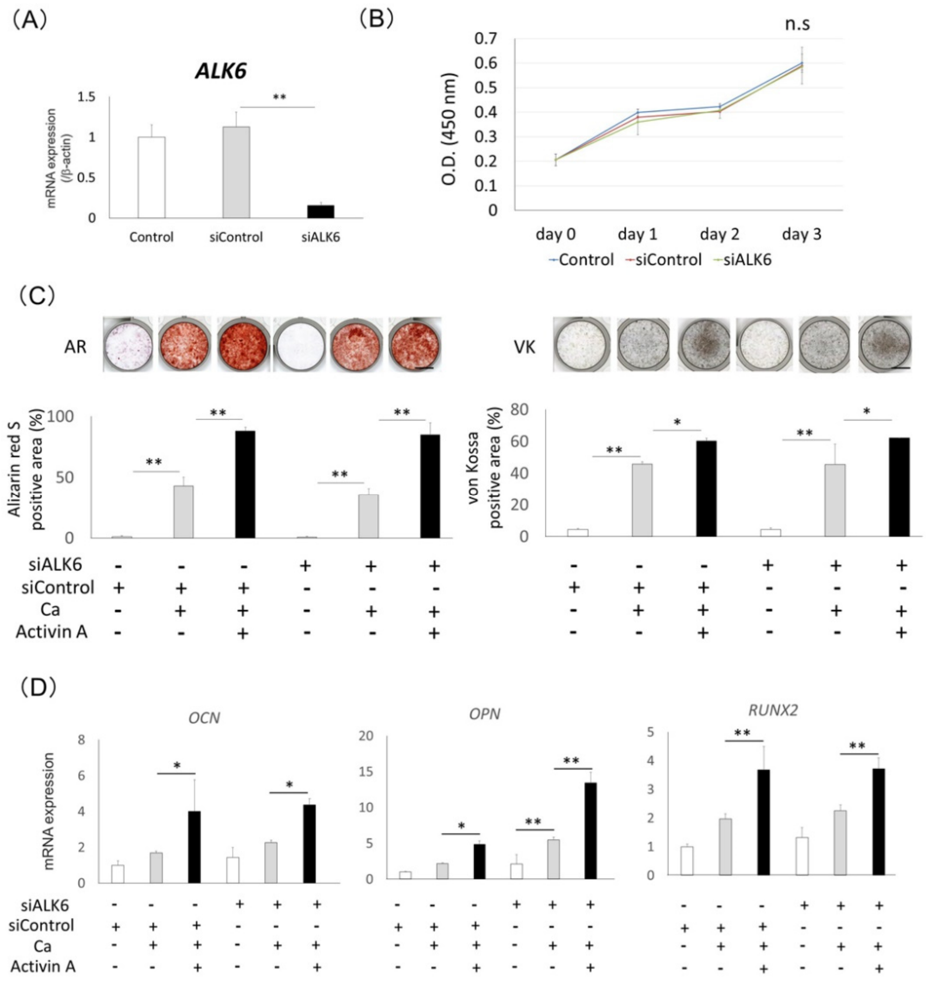

Supplemental Figure S7 Treatment with siALK6 had no effect on Activin A-induced osteoblastic differentiation of Saos2 cells

(A) Gene expression of *ALK6* in Saos2 cells treated with siALK6. Untreated cells were used as the control. Gene expression of *ALK6* was examined using quantitative RT-PCR. Gene expression levels were normalized against *β-actin* expression, and the results are shown as the fold-increase of the control. Values are the means ± SD from three independent experiments. **P < 0.01. (B) Saos2 cells treated with siALK6 were cultured in 10%FBS/αMEM for 0, 1, 2, and 3 days. A proliferation assay was performed using a WST-1 proliferation assay kit at an absorbance of 450 nm. (C) Saos2 cells were cultured with 0.5 mM $CaCl_2$ (Ca) with or without 100 ng/ml Activin A. Mineralization was visualized by Alizarin Red S staining (AR) and von Kossa staining (VK), and the staining area was quantitated. *P < 0.05, **P < 0.01. (D) Expression of bone-related genes (*OCN*, *OPN*, and *RUNX2*) in Saos2 cells treated with 100 ng/ml Activin A was examined by quantitative RT-PCR. Untreated cells were used as a control. The expression levels of these genes were normalized against *β-actin* expression, and the results are shown as the fold-increase of the control. *P < 0.05, **P < 0.01.

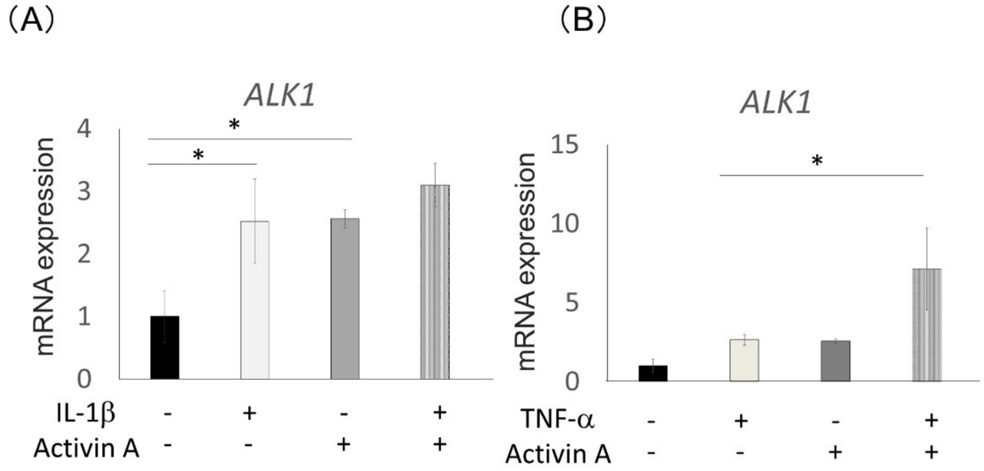

Supplemental Figure S8 The effects of Activin A on expression of *ALK1* in IL-1β- or TNF-α-treated Saos2 cells

(A, B) Gene expression of *ALK1* in Saos2 cells treated with IL-1β, TNF-α, or Activin A for 24 h was analyzed using quantitative RT-PCR. Untreated cells were used as the control. Normalization of gene expression was performed against *β-actin* expression, and the gene expression levels were shown as the fold-increase of the control. Values were the means ± SD from three independent experiments. *$P < 0.05$.

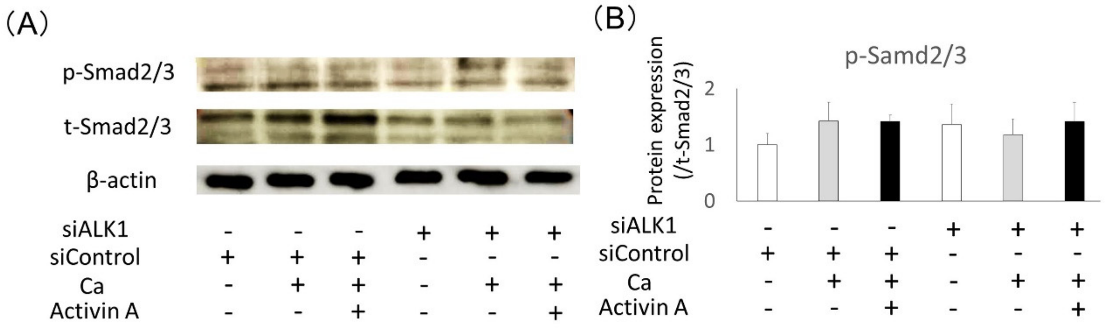

Supplemental Figure S9 Treatment with siALK1 exhibited no effect on phosphorylation of Smad2/3 in Saos2 cells

(A) Protein expression of phospho-Smad2/3 (p-Smad2/3) and total-Smad2/3 (t-Smad2/3) was analyzed using western blotting in Saos2 cells cultured with 0.5 mM $CaCl_2$ (Ca) with or without 100 ng/ml Activin A. β-actin was used as an internal standard for the control. (B) Expression levels of these proteins were normalized against t-Smad2/3 expression, and the results are shown as the fold-increase of the control. Values are the means ± SD from three independent experiments.
